# Medical Items and News of Interest to the Physician

**Published:** 1873-04

**Authors:** 


### Medical Items and News,

### For the Use of Physicians.

CULLED FROM THE STANDARD MEDICAL JOURNALS OF THE WORLD.

Note.—These formulæ are intended only for the regular practitioner of medicine, and should be employed only by him, or under his immediate supervision.

**Baker Brown.**

The death of this distinguished surgeon, of London, is announced.

**Dissections in Canada.**

After several ineffectual efforts, our Canadian friends have succeeded in securing a law legalizing dissections.

**Drs. Fordyce, Baker and T. Gaylard Thomas,** of New York, have been elected to honorary fellowship in the Obstetrical Society of London.

**Declined.**

Prof. Max Von Pettenkofer declines to leave Munich for Vienna, to the great disappointment of all connected with the school in the latter city.

**U. S. Dispensatory.**

A new edition of this work is in preperation, and will be issued in a few months. It will be in accordance with the recent changes in the nomenclature, etc., of the Pharmacopia.

**Yellow Fever at Rio de Janeiro.**

Telegraphic intelligence from Lisbon, dated Jan. 28, announce that the mail steamship from the above mentioned place, on the 11th inst., brought the news that yellow fever was raging in Rio, at the date of departure.

**Constipation.**

The *Phila. Med. Times* recommends the following in cases of constipation:

℞

Ext. physostigmæ ven., gr. iij;

Glycerinæ, f ℥ ij. M.

Sig:—Four drops four times daily.

**Chloral in Eczema.**

A correspondent of the *Boston Medical and Surgical Journal*, states that during the past year he has used, in several cases of chronic eczema, with very much satisfaction to his patients and himself, a solution of the hydrate of chloral as a topical remedy, in proportion of one or two drachms to a pint of water, applied two or three times a day.

**Cerebro Spinal Meningitis.**

Dr. F. P. Whitehead, Vicksburg, Miss. (*Richmond and Louisville Med. Jour.*), advocates the use of the fluid extract of gelseminum in this disease, and cites a case in which this remedy was very grateful to the patient.

**A Hæmorrhoidal Suppository.**

Dr. Henry M. Field, Newtown, Mass., (*Journ. Gynœcological Soc.*), recommends this formula for a suppository in hæmorrhoidal cases: ℞. Acid. tannic., gr. v.; Ext. belladona, gr. ss–i.; Butyr. Cacao, q. s.

**Test of Pure Air.**

Dr. Angus Smith gives a good rule for ascertaining the amount of carbonic acid in the air of houses: "Let us keep our rooms so that the air does not give a precipitate when a 10½ ounce bottleful is shaken with half an ounce of clear limewater; a sanitary regulation which is easily carried out."—*Med. & Surg. Reporter.*

**Spasm of the Glottis.**

A German medical journal says that Dr. Rehn treated a threatening case of spasm of the glottis, in which the attacks were very severe and frequent, with chloral-hydrate. The attacks were perceptibly lessened and finally quieted. The child thus treated was seven months old. The medicine was well borne, and produced no disturbances of digestion.

**Glycerine Ointment.**

Prof. Chas. A. Joy (*Jour. Applied Chemistry*), gives the following formula for a glycerine ointment, useful in chapped hands and excoriations: One half ounce of spermaceti is melted together with a drachm of white wax and two fluid ounces of oil of almonds, by moderate heat; the mixture is poured into mortar, when a fluid ounce of glycerine is added to it and rubbed until the ingredients are thoroughly mixed and cold.

**For Excessive Perspiration of Hands or Feet**

A German pharmaceutical journal recommends the following:

Carbolic acid, 1 part.
Burnt alum, 4 parts.
Starch, 200 parts.
French chalk, 50 parts.
Oil of lemon, 2 parts.

Make a fine powder, to be applied to the hands and feet, or to be sprinkled inside of the gloves or stockings.

**Arbor Vitæ in Small-pox.**

The leaves of *Thuja orientalis* and *occidentalis* are employed in Belgium against small-pox. A tincture is prepared by macerating, for ten days, one part of the fresh leaves with ten parts of ninety per cent. alcohol; it is given in water in doses of ten drops.—*Journ. de Pharm et de Chim.*

**Mouth Wash.**

The following is endorsed by good authority as excellent for the treatment of flabby, soft gums that bleed easily:

℞

Tinct. of Nutgalls, from ʒ ii to ℥ ss;

Water, O. j.

An ounce of tincture of orange-peel will improve the taste.

**Ingrowing Toe-Nail.**

Mr. Blower cures ingrowing toe-nail by first destroying with nitric acid any granulations that may be present, and then introducing beneath the nail a piece of finely compressed sponge, of a size something less than a grain of rice. The piece being retained in place by strips of adhesive plasters, applied longitudinally in order to avoid compression; the swelling of the sponge by imbibition causes the nail to be separated from the irritated flesh.—*British Med. Journal.*

**For Gastralgia.**

Prof. C. C. Cox recommends the following:

℞

Tinct. opii;

Tinct. capsici;

Æther sulph.;

Tinct. camphori, āā ʒ iij;

Chloroformi, ʒ j. M.

S. Teaspoonful, *pro re nata.*

The effacy of the above is much increased if taken in a wine-glass of hot ginger tea.

**Abortive Treatment of Boils and Whitlow.**

Dr. Simon de Forges (*Rev. de Therap.*) advises the topical use of camphorated spirits as an abortifacient in boils and whitlow. In the former case the boil is to be rubbed eight or ten times by the finger dipped in the alcohol. He asserts that it is rare that after this treatment a boil goes on further towards suppuration. In cases of whitlow he advises the patient to dip the finger for some ten minutes in camphorated spirits. This almost always gives great relief of the pain, and often cures the complaint.—*Medical Press.*

**Freckle Lotion.**

The following is commended by the *Druggists' Circular*, as a preparation for this purpose which does not contain mercury:

℞

Muriate of ammonia, ʒ j;

Distilled water, ℥ vij;

Cologne water, ʒ ij;

**Styptic Cotton.**

Dr. Rohland of New York, (*The Medical Record*), has prepared a styptic cotton (*Gossypium Stypticum*) that promises to be a useful addition to the means of arresting passive hemorrhages from extensive surfaces. It has the advantage of cleanliness and convenience, and possesses no irritating properties. It is prepared by boiling the cotton in a solution of alum and gum of benzoin, and, after the cotton is dried and picked, it is saturated by perchloride of iron. It is put up in boxes of convenient size.

**Pulmonary Hemorrhage.**

H. N. Eastman, M. D., of Geneva, N. Y. (*Buffalo Med. & Surg. Journal*), employs veratrum viride, in pulmonary hemorrhage when no acceleration of the heart's action is present, for the express purpose of reducing the normal circulation to the lowest point compatible with the safety of the patient, and to distend the pulmonary vessels as little as possible with the incoming blood. The idea is that this prevents further rupture of the capilliarys of the part, and affords an opportunity for the vascular lesions already existing to be obviated, by temporarily reducing the normal circulation.

**Asthma.**

The following is transcribed from the Receipt Book of the Philadelphia Hospital:

Belladonna leaves, grs. xcvj.
Hyoscyamus leaves,
Stramonium leaves, āā grs. xlviij.
Ext. opium, grs. iv.
Tobacco, grs. lxxx.
Boiling water, O.j.

Add

Potass. nit. grs. cxx.
Potass. arsenit. grs. cccxx.

Take thick bibulous paper; soak it in this solution, and allow to dry. When set on fire and the flame extinguished, this paper burns slowly without flame and emits a dense smoke which may be inhaled for the relief of asthma, often with very marked benefit. It is also useful in chronic bronchitis.

**Hops as an External Application.**

If possessed of any virtue as an anodyne, and popularly, hops have a great reputation as such, the effect is best obtained by *steaming* a muslin bag full of them, on the side to be applied to the body, until quite soft and damp—not wet.

The ordinary method of wringing out in hot water wastes all the strength of the hops, and makes a nasty mess of it.—*Boston Med. & Surg. Journal.*

**Hay Fever.**

Morrill Wyman, M. D., in his work on "Autumnal Catarrh," advocates in the asthmatic stage the smoking of Stramonium, also the espic cigarette. The composition of each cigarette is as follows:

℞

Belladonna leaves, gr. 4½;

Hyoscyamus. gr. 2¼;

Stramonium, gr. 2¼;

Phelandrium aquaticum leaves, gr. ¾;

Opium, gr. 1-5;

—*Med. Record.*

**Strychnia Poisoning.**

Dr. O'Farrell, of Philadelphia, writes to the *Phila. Med. Times*, that he was called to see a German æt 50, who had taken two grains of strychnia. He had already taken an emetic, so he gave him chloroform, in f ʒ doses, every half hour. He also administered tannin in five-grain doses, frequently repeated, with large doses of animal charcoal at intervals. After the chloroform had been given as above, for eighteen hours, he slept, and when he awoke, he expressed himself free from pain. The following day he was well and at work.

**Witness Necessary.**

A London correspondent of the *Dental Cosmos*, mentions the recent charge of indecent assault by Caroline Pope, a servant, against Jas. T. Grant, dentist, at the Jersey (Channel Islands) police court. She states that "gas" was given to extract a tooth, and while she was under its influence, he grossly outraged her. There was no evidence of rape having been attempted, and many witnesses gave the dentist the highest moral character. He was admitted to bail in the sum of £250 for future trial. This case illustrates once again, the extreme danger of administering anæsthetics to women without the presence of a witness. —*Medical Record.*

**Aperient and Alterative for Children.**

The *Georgia Medical Companion* commends this formula for children suffering from dyspepsia, with offensive breath, acid eructations, and constipation:

℞

Sodæ bicarbonatis, grs. xx.;

Tr. rhei, f. ʒ ij;

Infus. columbæ;

Decoc. taraxaci, āā f. ʒ vii. M.

S.—Two teaspoonfuls, to be taken night and morning. For a child one year old.

---

**Anatomical Specimens.**

The following is from French authority:

℞

Glycerine, ℥ xv;
Brown sugar, ℥ ijss;
Nit. potash, ℥ j. M.

After maceration for some days, the specimen becomes rigid, but recovers its suppleness on exposure to a dry, warm air. When dry they may be varnished. The time for maceration depends on the size of the specimen.

---

**Neuralgia.**

The *Georgia Medical Companion* contains the following formula:

℞

Ext. hyoscyamus;
Ext. valerian;
White oxide of zinc, āā ʒ j. M.

Make the mixture into sixty pills. One pill may be taken every three hours. This is an old and favorite prescription of the German and French physicians, and mentioned in many of their works on therapeutics.

---

**Expectorant Mixture.**

The *Journal of Materia Medica* states that the following recipe is very effective in treatment of coughs, colds, asthmatic difficulties, etc.:—

℞

Tinct. benzoin co., Cong. iss.;

Tinct. sanguinariæ;

Tinct. lobeliæ;

Tinct. opii camph.;

Syr. scillæ, āā Oiii.;

Ol. helianth. ann., ℥ v;

Ol. sassafras, ℥ ij;

Mellis. colat. opt. lbs. xii. M.

Dose, a dessert-spoonful every three or four hours, or more frequently if the cough is hard and expectoration difficult.

**Salve for the Lips, &c.**

The following is used for excoriations, warts and cracks, superficial ulcerations of the lips, mucous membrane of the cheeks, red parts of margin of nose, and other parts of the skin which are normally red:

℞

Ammoniated mercury, gr. vj.
Carmine, gr. i.
Soft cerate, ʒ ij.

Mix exactly.

The parts should be dried before the salve is applied.

---

**Odontalgia.**

Dr. A. L. Cory, in the *Chicago Medical Times*, reports that chloral is a remedy for toothache. Being called upon immediately after the fire to extract some aching teeth, and not having the necessary implements, and most of the dentists having lost their instruments, he prescribed 20 grains of hydrate of chloral, to lessen the pain and procure rest until he could obtain necessary forceps. To his astonishment, in every case, the ache not only promptly vanished, but failed to return. He has now tried this plan in about 20 cases with quite uniform success.

---

**Syrupus Cubebæ.**

A correspondent of the *Journal of Pharmacy* gives the following directions for preparing a syrup of cubebs, which has been found an elegant as well as efficacious remedy in diseases of the throat and lungs:—

Fld. ext. cubebs, f ℥ ij;
Carb. magnesia, f ℥ ss;
Sugar, powdered, ℥ xij;
Orange-flower water, f ℥ ij;
Water, q. s.;
Ess. oil almonds, gtt. j.

Rub up the fld. ext. with the carb. magnesia and then add ℥ ij. of the sugar in small portions. When thoroughly mixed, add gradually first the orange-flower water and then f ℥ vij. water, constantly triturating the mixture until the sugar is dissolved. Filter and add q. s. water through the filter to measure f ℥ xj., in which dissolve the balance of sugar without heat. Add the oil of almonds, put in a little alcohol, and again filter, adding, if necessary, q. s. water through the filter to measure 1 pt. The dose of this syrup is f ʒ j-iv., and it may be given in even larger doses if desired.

**Collodion with Sesquichloride of Iron.**

℞

Sesquichloride of iron, ℥ j ;
Stronger alcohol, f ℥ x ;
Stronger ether, f ℥ xij ;
Soluble cotton, ʒ j ;

Into a suitable bottle introduce the cotton, pour on two fluid ounces of alcohol, and shake well, then add the ether, and agitate frequently until dissolved. Dissolve the sesquichloride of iron in the balance of the alcohol, mix with the prepared collodion.—*Am. Jour. Phar.*

---

**Local Anæsthetic.**

J Knox Hodge, M. D., (*Geo. Med. Companion*), recommends the following formula as a local agent of signal potency in all neuralgic affections, and for the relief of pain generally:

℞

Albumen of egg, ʒ j ;

Rigoline, ʒ iv ;

Oil of peppermint, ʒ ij ;

Collodion,

Chloroform, āā ʒ j ; M.

Agitate occasionally. Apply by smart friction with the hand, or generally with a camel's-hair pencil along the course of the nerve involved.

---

**Strumous Ophthalmia.**

Dr. James Braithwaite, of Leeds, writes to the *Practitioner*, that he has for many years treated this affection by the internal use of belladonna, given commonly in the form of the extract in an eight ounce mixture containing four grains, and a half drachm of iodide of potassium. Of this, the dose for a child two years old, is a teaspoonful every four hours. If no improvement results in a few days, the dose may be increased somewhat, and the quantity of belladonna in the mixture increased to five grains, and the iodide to a drachm.—He believes that belladonna should be given at once, without waiting until other means fail; that its efficacy is very much enhanced by the combination with the iodide of potassium, and prefers to use the extract of belladonna smeared upon the eye-lid, eye-brow, and temple, to the application of atrophene to the ball, since the latter is apt to increase the irritation and photophobia instead of relieving it. He advises the use of cod-liver oil, and finds change of air and counter irritation with iodine paint, sometimes useful in the latter stages. Iron, he thinks, is injurious.

**Chronic Bronchitis.**

The *Georgia Medical Companion* gives the following as Dr. Horace Green's formula :

℞

Acidi hydrocyanici medicinalis, gtt. 40.
Morphiæ sulph. gr. iij.
Tinct. sanguinariæ
Vini ipecac, āā f ℥ ss.
Syr. pruni virginianæ vel mistura amygdal, f ℥ v.

S. A teaspoonful twice a day.

Dr. Green has found this a most valuable remedy in all pulmonary catarrhal diseases unattended with fever. As the acid is apt to float on the top of the liquid, the vial should be shaken on the administration of each dose.

---

**Lice.**

W. T. Carter, M. D., Louisville, Ky., (*Am. Practitioner*), recommends the following solution for the destruction of parasites :

℞

Corrosive sublimate, gr xij;

Alcohol, ʒ iv ;

Ol bergamot, ♏ vj ;

Mix and add water, ℥ iij ss.;

This mixture should be thoroughly applied to every part of the body infested. The first application will, in the majority of cases, cause the death of every accessible louse; but it should be continued twice daily for at least one week, in order that none may escape.

---

**Removing a Foreign Body from the Nose.**

"Ranking's Abstract" relates the case of a child from whose nose surgeons failed to remove a cherry-stone, and were out-done by the village barber, who administered an emetic, and, at the moment when vomiting was about to commence, clapped a handkerchief tightly over the mouth of the child. A correspondent of the *Atlanta Medical Journal*, tells quite as good a story of a Southern practitioner, who was called to see a child with a foreign substance in its nostril, which had held its position in spite of efforts for its removal directed by the professional skill of "all the region round." On the way, the doctor was saluted by an aged negro woman, who asked him if he was going to see that child.—On receiving an affirmative answer, she said, "Put yer finger 'long side the nose, t'other side from the thing, and with yer own mouf over the child's mouf, blow hard, and it's bound to come out." He followed her directions, and the result was as she had predicted.

**Carbolic Acid in Small-Pox.**

Dr. A. Loffier states, in a Vienna medical journal, that he has treated more than forty cases of small-pox by the external copious application, by means of cotton wool, of a solution of one part of carbolic acid in twelve of oil. The result in all the cases was, that the cutaneous swelling soon diminished; and that when the application was made early, the course of the disease, in relation to the number of pustules, was milder. He believes, also, that by this treatment, the danger of infection was greatly diminished.

---

**Styptics.**

In an inaugeral essay, Dr. Chas. H. Mitchell, (*American Jour. Pharmacy*), presents the following new formulæ:

℞
Tannin, ℥ ij;
Stronger alcohol, f ℥ iv;
Stronger ether, f ℥ xij;
Soluble cotton, ʒ j;
Canada balsam, ʒ j;

Introduce the cotton into a suitable bottle, pour on it two fluid ounces of alcohol, shake well; then add ten fluid ounces of the ether, agitate frequently until dissolved. Dissolve the tannic acid in a mixture of the remainder of the alcohol and ether, mix with the first liquid, add the balsam, allow to stand until clear; then pour off.

---

**For Colds and Catarrh.**

A Berlin correspondent of the London *Druggists' Circular*, recommends the following as an effective, simple, and not unpleasant remedy for these troublesome affections:

A wide-mouthed, glass-stoppered bottle is filled with cotton, which is then saturated with this mixture:

℞

Acidi carbolic; puriss. ℈ iv.

Liq. ammon. caustic., sp. gr. 0.960, ʒ iss.

Aquæ destillat., ʒ ij. ℈ ij.

Spirit. vini rectificatiss, ℈ iv.

The vapors are drawn into the nose frequently during the day, and now and then inhaled into the mouth. A medical gentleman of Stettin, who is renowned not only for his skill as a physician, but likewise for the tremendous catarrh that troubles him every winter, has used this *olfactorium anticatarrhoicum* with perfect success on his own person, and afterwards on many of his patients, and recommends it highly.

**Cirrhosis and Albuminuria.**

In a case of coexistence of albuminuria and cirrhosis, published in the *Chicago Med. Examiner*, the following prescriptions were recommended by Dr. N. A. Davis:

℞
Tinct. digitalis,
Fld. ext. lactuca,
Syrp. prunns virg., āā ℥ ij;
Iod. patass., ℥ iij; M.

Take one teaspoonful every six hours.

℞
Spts. nit. dulc., ℥ iij;
Tinct. ferri chl., ℥ ij; M.

Take one teaspoonful every six hours, alternating it with the other prescription.

---

**Caffeine.**

Dr. H. S. Purdon has lately been trying the hypodermic injection of caffeine in neuralgia from both functional and organic causes.—Among his patients at the Belfast General Hospital, was a case of "central neuralgia," which had resisted all plans of treatment. Under the daily use of caffeine hypodermically, commencing with half-grain doses, and increasing to one grain (dissolved in tepid water), very satisfactory results were obtained. Caffeine is superior to morphine in never producing sickness, which is so common where the latter is used, hypodermically in any considerable dose. This salt has proved serviceable in allaying the sleeplessness of delirium tremens.—*The Doctor.*

---

**Anthrax.**

The French medical journal, *Le Movement Medical*, has the following: "We believe that the most effective treatment of anthrax is by crucial incision. It may also be opened subcutaneously, an operation less painful, than the preceding. For timid patients, the following proceeding is recommended: The canula of a hypodermic syringe is introduced into the centre of the tumor, and pus, if present, will rise on drawing the piston. The syringe is detached and emptied, leaving the canula in place, it is again re-adjusted to the canula and the piston drawn as long as pus continues to rise; when the pus is completely evacuated, the canula is taken out and the following preparation applied to the tumor:

℞
Sul. æther;
Glycerine, āā f ℥ j;
Carbolic acid, (cryst.) gr. v. M.

Sig: Apply with camel's hair brush.

**Myrrh in Dyspepsia.**

Dr. Delioux de Savignac (*Bull. de. Therap.*, Dec. 15, 1871), writing on the therapeutic properties of myrrh, observes: "The stomach is the portion of the digestive apparatus which has appeared to me to be most happily influenced by myrrh. My experience fully confirms the reality of the stomachic properties which have been attributed to it by the older writers. I have seen painful forms of dyspepsia rapidly relieved, and even cured, by the use of this gum-resin; sometimes I have given it in conjunction with other remedies, such as bismuth, magnesia, bicarbonate of soda, etc., drugs which have hitherto proved ineffectual, and sometimes I have employed it alone, so as to leave no doubt as to the efficacy of its action."

**Gelseminum.**

We notice various communications to the medical journals relative to the use of gelseminum in malarial fevers. In remittent fevers, Dr. John S. Hughson, of S. C., uses gelseminum, eight drops every three or four hours during the paroxysm, as well as in the remission, adding quinine in the remission. After cessation of fever he gives gelseminum alone. In intermittent fever, he prescribes it in eight to ten drops (Tilden's Fluid Extract), four times a day, until convalesence is beginning to be established, then three times daily. In chronic cases of intermittent fever, he combines it thus:

℞

Liq. potass. arsen.;

Fld. ext. gelsem., āā, ℥ ss. M.

Sig: Take sixteen drops, just after each meal, for one week; then omit for five days, take again for two days, continuing so for one month.

**Summary Therapeutics.**

A student at Guy's Hospital writes to the *Medical Gazette* as follows: "One night, going round the wards, I found a girl in a fit; she fought and struggled with her nurses. Hating all rows, I grasped her throat and held it firmly till the astonished girl gasped out, "you are choking me." I then relaxed my hold, and promised her a repetition of the performance. She was thoroughly cowed after having two more fits, and being twice nearly choked; her alarm was so great that, though every night previously for months past she had had fits, she desisted, and a month afterward her mother told me with joy of her complete recovery. A few days later, Mr. Stocker, whose experience is immense, told me how beneficial this plan was, and said, "you may remain for hours fighting with a screaming girl, but carefully choke them and they immediately subside; it is unpleasant for them, it frightens them."

**Colds.**

Dr. Dobell, in his recent work on winter cough says, in emphatic italics, "*colds can be stopped without lying in bed, staying at home, or in any way interfering with business.*" He says that his plan, if begun directly the first signs of catarrh show themselves in the nose, eyes, throat and chest, is almost infallible, but will not answer if the cold has become thoroughly established. The plan is as follows:

"Give five grains of sesquicarbonate of ammonia, and five minims of liquor morphiæ, in an ounce of almond emulsion every three hours. At night, give ℥ iss. of liquor ammoniæ acetatis in a tumbler of cold water, after the patient has got into bed and been covered up with several extra blankets; cold water to be drank freely during the night, should the patient be thirsty.

In the morning, the extra blankets should be removed so as to allow the skin to cool down before getting up.

Let him get up as usual, and take his usual diet, but continue the ammonia and morphia mixture every four hours. At bedtime, the second night, give a colocynth pill. No more than twelve doses of the mixture from first to last need be taken, as a rule; but should the catarrh seem disposed to come back after leaving off the medicine for a day, another six doses, and another pill may be taken. During the treatment the patient should live a little better than usual, and on leaving it off, should take an extra glass of wine for a day or two. Dr. Dobell says his patients call this the "magic mixture."—*Boston Med. & Surg. Jour.*

**A Sovereign Anti-Billious Pill.**

The *Georgia Medical Companion* says; We transcribe (by permission) from the Prescription Book of one who has been actively engaged in practice for the last thirty years, a formula for an Anti-Billious Pill, which, we feel confident, will find favor with all disposed to adopt it. As will be seen, besides containing all the components of the "Cook Pill,"—

a pill of established popularity in the South,—it also embodies other important adjuvants. The gamboge and podophyllin give increased action and augmented hepatic tendency, whilst the capsicum and extract hyosciamus impart to the whole a fine corrective and anti-drastic quality. By substituting pure castile soap for the calomel, we also have a pill that will effect all that is meant by a "Vegetable Anti-Billious Pill:"

℞

| | | |
|---|---|---|
| Calomel, Rhubarb, Aloes, | āā | dr. j. |
| Capsicum, Camboge, Podophyllin, Ext. Hyosciamus, | āā . . | gr. xij. M. |

Ma e mass with Ar. Syr. Rhubarb, and divide into 22 pills.

Dose from 2 to 4 for adults, as a prompt and efficient anti-billious evacuant.

---

**Post Mortem of Napoleon III.**

The examination of the body of the Emperor took place on Jan. 10th, and occupied two hours. The following is the official report: The most important result of the examination was that the kidneys were found to be involved in the inflammatory effects produced by the irritation of the vesical calculus. The disease of the kidneys was of two kinds. There was, on the one hand, dilatation of both ureters, and of the pelves of both kidneys. On the left side the dilatation was excessive, and had given rise to atrophy of the granular substance of the organ; on the other there was subacute inflammation of the uriniferous tubes, which was of more recent origin. The parts in the neighborhood of the bladder were in a healthy state; the mucous membrane of the bladder and prostatic urethra exhibited the signs of subacute inflammation, but not the slightest indication of injury. In the interior of the bladder was found a part of a calculus, the form of which indicated that half had been removed. Besides this there were two or three extremely small fragments, none of them larger than a hemp seed. This half-calculus weighed about three-quarters of an ounce, and measured one-quarter of an inch by fifteen-sixteenths of an inch. There was no disease of the heart, nor of any other organ, excepting of the kidneys. The brain and its membranes were in a perfectly natural state. The blood was generally liquid, and contained only a very few small clots. No trace of obstruction by coagula could be found either in the venous system, in the heart, or in the pulmonary artery.

Death took place by the failure of the circulation, and was attributable to the general constitutional state of the patient. The disease of the kidneys, of which this state was the expression, was of such a nature, and so advanced that it would in any case have shortly determined a fatal result."

Signed, J. Burdon Sanderson, M. D.

Dr. Conneau,

Dr. LeBaron Corvisart,

Henry Thompson,

J. T. Clover,

John Foster.

Camden Place, Chiselhurst, Jan. 10, 1873, 6:30 P. M."

---

**Xylol in Small-Pox.**

Dr. Nagel says: "I have administered xylol in eighty-one cases of small-pox; among these were eight children under two years of age—six of them not vaccinated; eleven children from two to ten years of age—four of them not vaccinated; twenty patients under twenty-five years of age; the others were above that age, and one was aged sixty. Of all these eighty-one patients (thirty-four of whom had the small-pox in its worst form), only four died—one child, three weeks old, not vaccinated; another child, nine months old, not vaccinated, and two adults of about thirty-two years of age. I conclude, therefore, that,

1st. Xylol produces positively better effects in the treatment of small-pox than any other remedy known.

2d. Xylol positively mitigates the severity of the disease, accelerates the recovery, and prevents the hitherto great mortality.

3d. Xylol, administered when a contagion is suspected, and before the eruption of the disease, does not prevent the breaking out of small-pox, but facilitates the elimination of the disease.

Under these circumstances, I can do no less than most warmly recommend the administration of xylol.

The dose of xylol, as a prophylactic, is ten to fifteen drops once a day. During the progress of the disease, the same dose is given four times a day. As much as a teaspoonful has been taken at a time without serious results. It is administered in wine, syrup or water. Raspberry syrup is especially recommended.

